# Books and Magazines

**Published:** 1906-03

**Authors:** 


### Books and Magazines.

Thornton's Pocket Medical Formulary (heretofore known as The Medical News Pocket Formulary) new (7th) edition, revised to accord with the new U. S. Pharmacopeia, containing more than 2000 prescriptions with indications for their use. In one leather-bound volume. Price, $1.50, net. Lea Brothers & Co., Publishers, Philadelphia and New York, 1906.

The changes in the new Pharmacopeia were many and important. Some medical preparations were nearly doubled in strength and their doses proportionately reduced; others were much weakened and doses increased. The new edition of Dr. Thornton's Formulary is revised not only to include all the newer drugs, but also to accord with the new Pharmacopeia, appears most timely, and its importance is obvious.

---

TRIP TO THE "LAND OF THE MIDNIGHT SUN." By Dr. Flavel B. Tiffany. Hudson Press, Kansas City, Mo., 1905.

A most delightful booklet description in a most charming style of the author's visit to the Land of the Midnight Sun. The author is a well-known specialist of Kansas City, Mo.

---

WILLIAMS ON FOOD.—FOOD AND DIET IN HEALTH AND DISEASE.—A MANUAL FOR PRACTITIONERS OF MEDICINE, STUDENTS, NURSES, AND THE LAY READER. By Robert F. Williams, Professor of Principles and Practice of Medicine in the Medical College of Virginia, Richmond. In one handsome 12mo volume of 392 pages. Cloth, net, $2. Lea Brothers & Co., Publishers, Philadelphia and New York, 1906.

Doctors will welcome this book as one which they can recommend to their patients as a guide to the preparation and use of food in sickness and convalescence. For mothers the book will be especially valuable. Ignorance is always costly. This is particularly true in the feeding of growing children, in whom habitual errors of feeding frequently produce effects lasting through life as well as temporary illness.

Nurses and hospital superintendents will find an attractive feature of the work in the last section, where is given a great number of recipes for foods for sick patients and convalescents, with full directions for preparing and administering.

---

ESSENTIALS OF MATERIA MEDICA, THERAPEUTICS, AND PRESCRIPTION WRITING. By Henry Morris, M. D., College of Physicians, Philadelphia. Seventh thoroughly revised edition, adapted to the new (1905) Pharmacopeia. By W. A. Bastedo, Ph. G., M. D., Instructor in Materia Medica and Pharmacology at the Columbia University (College of Physicians and Surgeons), New York City. 12mo, 300 pages. Philadelphia and London: W. B. Saunders & Co., 1905. Cloth, $1, net.

The student can not find a better or more practical work on Materia Medica, Therapeutics, and Prescription Writing than this little essentials from the press of W. B. Saunders & Company. But then, this work is no exception in this respect to all the other numbers of this excellent series of compends. Dr. Bastedo, in revising the book for this seventh edition, has brought it in accord with the new (1905) Pharmacopeia, introducing all the new remedies and carefully indicating their therapeutic doses and uses. For a work of three hundred pages it contains a mine of information so presented as to be easily grasped. We give it our unqualified endorsement.

